# Developmental trajectories of sensitivity to threat in children and adolescents predict larger medial frontal theta differentiation during response inhibition

**DOI:** 10.1093/scan/nsad009

**Published:** 2023-03-03

**Authors:** Taylor Heffer, Stefon van Noordt, Teena Willoughby

**Affiliations:** Department of Psychology, Brock University, St. Catharines, Ontario L2S 3A1, Canada; Department of Psychology, Brock University, St. Catharines, Ontario L2S 3A1, Canada; Department of Psychology, Mount Saint Vincent University, Halifax, Nova Scotia B3M 2J6, Canada; Department of Psychology, Brock University, St. Catharines, Ontario L2S 3A1, Canada

**Keywords:** sensitivity to threat, theta power, EEG, stability over time, response inhibition

## Abstract

Sensitivity to threat (ST) is thought to be a hallmark of the onset and maintenance of anxiety, which often manifests behaviorally as withdrawal, increased arousal and hypervigilant monitoring of performance. The current study investigated whether longitudinal trajectories of ST were linked to medial frontal (MF) theta power dynamics, a robust marker of performance monitoring. Youth (*N* = 432, *M*_age_ = 11.96 years) completed self-report measures of threat sensitivity annually for 3 years. A latent class growth curve analysis was used to identify distinct profiles of threat sensitivity over time. Participants also completed a GO/NOGO task while electroencephalography was recorded. We identified three threat sensitivity profiles: (i) high (*n* = 83), (ii) moderate (*n* = 273) and (iii) low ( n= 76). Participants in the high threat sensitivity class had greater levels of MF theta power differentiation (NOGO-GO) compared to participants in the low threat sensitivity class, indicating that consistently high threat sensitivity is associated with neural indicators of performance monitoring. Of concern, both hypervigilant performance monitoring and threat sensitivity have been associated with anxiety; thus, youth with high threat sensitivity may be at risk for the development of anxiety.

Sensitivity to threat (ST), a heightened responsiveness to aversive situations, is thought to be an important factor associated with anxiety (Gray, 1970, 1982; Gray and McNaughton, 2000). Yet not all youth who are sensitive to threat develop anxiety. There is increasing evidence suggesting that cognitive control may be an important factor to consider when assessing ST and that examining both cognitive control and ST may help distinguish which youth may be most at risk (e.g. Fox *et al.*, 2021; Valadez *et al.*, 2021). Indeed, some aspects of cognitive control, including hypervigilant performance monitoring and overcontrolled behavior, are thought to be important factors that maintain anxiety-related problems, such as heightened threat sensitivity (White *et al.*, 2011; Lamm *et al.*, 2014). The current study investigated whether medial frontal (MF) theta dynamics, a neural marker of performance monitoring, is associated with longitudinal trajectories of ST in children and adolescents.

## Threat sensitivity and performance monitoring

Concurrent levels of anxiety/threat sensitivity have been associated with hypervigilant performance monitoring (Gray and McNaughton, 2000; Grillon *et al.*, 2017). For example, individuals with higher levels of anxiety perform better on inhibitory control tasks (when there is a need to inhibit their natural or dominant response) than individuals with lower levels of anxiety (e.g. Grillon *et al.*, 2017; Iijima *et al.*, 2019; Troller-Renfree *et al.*, 2019). Similarly, several studies have shown that response inhibition is associated with higher levels of behavioral inhibition (McDermott *et al.*, 2009; White *et al.*, 2011), a related concept to ST, characterized by avoidant coping styles and attentional bias to threat (e.g. Perez-Edgar and Fox, 2005; Barker *et al.*, 2019). It may be that youth who are better at inhibiting behavioral responses may inappropriately withdraw from situations and this type of behavior may facilitate anxiety tendencies (Fox *et al.*, 2021). This idea is supported by the work of Crowley *et al.* (2021), who found that adolescents with high levels of behavioral avoidance had significantly higher scores on anxiety than adolescents with lower levels of avoidance behaviors.

In addition to behavioral findings, neural measures associated with cognitive control have been associated with threat sensitivity/anxiety (see van Noordt and Segalowitz, 2012). For example, the NOGO N2, an event-related potential (ERP) associated with cognitive control, is larger among anxious compared to non-anxious participants (Righi *et al.*, 2009; Hum *et al.*, 2013). Lamm *et al.* (2014) also found that behavioral inhibition during a GO/NOGO task was associated with performance accuracy, larger N2 activation, and greater dorsal anterior cingulate cortext/dorsolateral prefrontal cortext activation. These results point to overcontrol tendencies relating to anxiety-related problems.

## Threat sensitivity and MF theta

MF theta may be an important neural mechanism to consider when investigating performance monitoring and ST. MF theta-band oscillations are a robust marker of performance monitoring and conflict detection (Cavanagh and Frank, 2014; Oehrn *et al.*, 2014; van Noordt *et al.*, 2016). For example, MF theta is sensitive to conflict detection during tasks that involve competing response conflicts (Oehrn *et al.*, 2014) and behavioral adjustments following error commissions (Kalfaoğlu *et al.*, 2018).

MF theta is also sensitive to aversive conditions. For example, (Cavanagh *et al.*, 2012) found larger MF difference scores for conflict (high conflict–low conflict), punishment (punishment–reward) and error (error–correct) trials (see also Mueller *et al.*, 2014). Similarly, a meta-analysis by Cavanagh and Shackman (2015) demonstrated that individuals with higher anxiety-related problems had larger difference scores of MF theta in response to conflict, punishment and errors than individuals with lower anxiety-related problems. Cristofori *et al.* (2013) found significantly higher MF theta during exclusion, and other studies have found that increased theta to social exclusion is related to rejection distress (see also van Noordt *et al.*, 2015). MF theta also was larger for negative versus positive feedback and errors versus correct responses (e.g. Cohen, 2011; van de Vijver *et al.*, 2011). Thus, these differences in MF theta between conditions may reflect an important mechanism associated with threat sensitivity.

Investigating longitudinal trajectories of threat sensitivity in particular could identify the establishment and/or maintenance of threat sensitivity as it relates to MF theta dynamics. Degnan and Fox (2007) suggested that consistently highthreat sensitivity over time may be a good indicator of those who are most at risk for anxiety. In other words, an individual who has consistently high threat sensitivity over time would be at greater risk than an individual who reports consistently low or inconsistent threat sensitivity over time. Although no study has investigated trajectories of threat sensitivity in relation to MF theta dynamics, consistently high threat sensitivity has been associated with greater vulnerabilities, such as heightened avoidance motivations (Degnan and Fox, 2007; Heffer and Willoughby, 2020b).

## The current study

To address whether trajectories of threat sensitivity differ in MF theta difference score, we will first replicate the identification of distinct classes of threat sensitivity over time outlined by Heffer and Willoughby (2020b) with a larger sample (432 participants compared to 361 participants). We will then be able to investigate whether these distinct classes of threat sensitivity (e.g. consistently high threat sensitivity over time) are associated with heightened MF theta condition differences (NOGO-GO) during response inhibition. Given that this is the first study to assess MF theta dynamics and longitudinal trajectories of threat sensitivity among children and adolescents, our analysis is exploratory. However, we expect that individuals classified by consistently higher sensitivity to threat will have greater MF theta difference scores than individuals characterized by lower or inconsistent threat sensitivity. Given that latent class analyses assume that every participant in a given trajectory class has the same degree of threat sensitivity, we also will examine threat sensitivity trajectories continuously to assess the replicability of the pattern found when assessing distinct classes.

## Method

### Participants

Participants (*N *= 467, *M*_age_ = 10.60, s.d. = 1.80; age range = 8–14, 50.1% women) were drawn from several elementary and high schools in southern Ontario, Canada. Parent report indicated that 84.95% of the children and adolescents were White, 2.30% were Hispanic, 2.30% were Black, 1.28% were Asian, 1.02% were Indigenous and 7.40% were mixed (an additional 0.77% of parents indicated that they preferred not to answer the question). The average parental education in the sample was ‘completed an associate degree or diploma’ (*M* = 4.14, s.d. = 0.84). Students were part of a larger study examining the relationship between well-being and youth health-risk behaviors over time. The current sample completed surveys annually across 3 years and completed a mobile lab session where electroencephalography (EEG) was recorded during a GO/NOGO task, starting in Year 2 of the study. Parents/guardians were asked to identify whether their child had any illnesses or disabilities (either physical or mental). Three participants were excluded because of a diagnosis of autism (*n* = 1), cerebral palsy (*n* = 1) or concussion (*n* = 1). Thirty-two participants were excluded from the analyses due to recording issues (*n* = 10), the fact that they did not follow instructions (*n* = 3), excessive artifacts (e.g. movement/muscle artifacts) identified during preprocessing (*n* = 17), or outliers due to extreme values for theta power (*n* = 2). The final sample resulted in 432 participants. Excluded individuals did not differ from included participants on age, sex or level of parental education (proxy for socioeconomic status (SES)), *P* > 0.05.

### Procedure

Students were invited to participate in the larger study through visits to schools. Surveys were completed in classrooms during school hours, and all participants received gifts (e.g. backpacks) as compensation. Participants also completed a Mobile Lab component during which EEG data were recorded. The study was approved by the University Ethics Board. Participants provided written informed assent, and parents/guardians provided written informed consent.

### Missing data analysis

Missing data occurred because some children and adolescents did not complete all the questions in the survey (average missing data were 4.33% across the 3 years) or because some participants were not at school or in the classroom during the survey sessions (6.7% in Year 1, 4.9% in Year 2 and 13.0% in Year 3). Missing data were not only primarily due to absenteeism but also occasionally due to time conflicts, students declining to participate in the survey, research assistant errors (e.g. not inviting a child to complete the survey) or students moving to another school district with no contact information. Results of logistic regressions indicated that older age (*P* = 0.023) predicted missingness at Year 2. Older age likely predicted missingness because high school students sometimes have a spare period during the time that the survey is conducted and thus are not available to do the survey and generally are more likely than elementary school students to be absent from school (https://www2.ed.gov/datastory/chronicabsenteeism.html). Missing data were imputed using the expectation–maximization (EM) algorithm in SPSS, with 25 iterations, the default value. Age, sex, SES and behavioral inhibition scale (BIS) at each year were included in the imputation model. EM algorithm retains cases that are missing survey waves and thus avoids the biased parameter estimates that can occur with pairwise or list-wise deletion (Schafer and Graham, 2002).

### Measures

#### Sensitivity to Threat

At Years 1–3, participants reported the extent to which they agreed with three items specifically examining ST from the BIS (Carver and White, 1994) on a scale ranging from 1 (*strongly disagree*) to 4 (*strongly agree*). Higher scores indicate higher levels of threat sensitivity. Cronbach’s alpha was 0.77, 0.80 and 0.80 at Years 1–3, respectively. Note that the original BIS item is ‘Criticism and *scolding* hurts me quite a bit’; however, pilot testing indicated that young children did not understand the term scolding, and therefore, we dropped that word from the question. The three items in our scale are thought to reflect BIS, and in our scale, these items are strongly correlated (range = 0.70–0.76) and hang together in a factor analysis (all factor loadings > 0.82).

#### GO/NOGO task

Participants completed a GO/NOGO task (DuPuis *et al.*, 2015; Heffer and Willoughby, 2020a) while EEG was recorded. Participants were instructed to continuously push a button every time a stimulus, a cartoon character, appeared (a GO trial) unless the current stimulus matched the previously presented stimulus (i.e. the same cartoon character appeared twice in a row), in which case the participant needed to refrain from pushing the button on that trial (an NOGO trial). A total of 225 trials were completed: 150 GO trials and 75 NOGO trials. Each stimulus was presented for 500 ms with an initial starting interstimulus interval (ISI) of 250 ms. The ISI dynamically adjusted throughout the task based on participants’ accuracy on the three most recent preceding NOGO trials, adding 15 ms to the ISI if performance was low (one or zero correct inhibitions on three preceding NOGO trials) or subtracting 15 ms from the ISI if accuracy was high (correct inhibitions on all three previous NOGO trials), to a minimum ISI of 250 ms and a maximum ISI of 1500 ms. The average ISI time duration was 646.09 ms, with a s.d. of 86.53 ms. No participants performed well enough to reach the minimum ISI (minimum ISI duration = 310 ms) or poorly enough to reach the maximum ISI (maximum ISI duration = 1045 ms).

### Electrophysiological recording

EEG was recorded continuously from a BioSemi ActiveTwo system using a 96-channel montage and 7 face sensors. The data were digitized at a sampling rate of 512 Hz. Preprocessing was conducted to identify channels, independent components and time periods with unreliable signals and high levels of relative non-stationarity.

### Preprocessing

Preprocessing was performed using the EEG Integrated Platform Lossless pipeline (EEG-IP-L) to identify channels, independent components and time course activity that contained artifacts and relative non-stationarity (see Desjardins *et al.*, 2021 for full details on this pipeline; see Heffer and Willoughby, 2020a; van Noordt *et al.*, 2022 for the current sample). To minimize spatial bias introduced by variance in channel artifacts across participants during the preprocessing, we used an interpolated average reference. Channels containing clean signals are used to interpolate to 19 spatially balanced sites arranged in 10–20 layouts. The average of these 19 interpolated sites is used as the reference and subsequently subtracted from each of the original channels containing clean signal. This re-referencing occurred following the flagging of channels that showed unreliable correlations with neighboring channel activity and following the flagging of bridge channels. At the final stage of processing, removed channels were interpolated using spherical spline and the full montage was re-referenced to the average of all channels.

EEG-IP-L uses a series of criteria functions to assess data quality, including signal variance, nearest neighbor correlations and independent component analysis to separate stable biological artifacts (e.g. heart rate components, eye blinks and electromyography) and sources of non-stationarity from cortical field projections. Components were classified using the ICLabel plugin (Pion-Tonachini *et al.*, 2019a, 2019b) which assesses each component against a crowd-sourced database to identify activation consistent with five different categories: eye blinks, neural, heart, lateral eye movements, muscle contamination and mixed signal (a combination of two or more categories listed).

After preprocessing, quality control review was completed by a trained research assistant to validate signal quality assessments based on component topographical maps, continuous activation, residual variance dipole fit and power spectrum profile. This method has been shown to increase data retention without impacting the robustness of ERP effects (Desjardins *et al.*, 2021).

### EEG post-processing and time-frequency decomposition

EEG data were then segmented into single trials and time-locked to the onset of GO and NOGO stimulus (−1000 to 2000 ms) from the GO/NOGO task. A final quality check was completed to identify and remove single channels which revealed temporally isolated extreme voltage fluctuations (±50 mV). Channels that were removed during preprocessing were interpolated (i.e. rebuilt using the remaining channel data) to the full montage of 103 channels (96 scalp and 7 exogenous) using spherical spline. There was an average of 113 GO (s.d. = 16.93, min = 33, max = 140) and 47 NOGO trials (s.d. = 10.21, min = 15, max = 69) available for analysis.

The single-trial data were convolved using complex Morlet wavelets to generate time-varying estimates of spectral power. The EEGLAB *newtimef* function was used to extract spectral power, using one cycle at the lowest frequency and increase in 0.05 Hz increments to the highest frequency. Spectral power was calculated for 27 linearly spaced frequencies from 3 to 30 Hz. Given that event-related theta dynamics have been found to increase from childhood to adolescence (Liu *et al.*, 2014), spectral power was baseline normalized using the −200 to 0 ms prestimulus window to account for the 1/*f* power scaling and remove activity in the signal that was not constant over time.

We focused on frontal midline channels as our region of interest (ROI), given the well-established increase in MF theta during inhibitory control. As described by van Noordt *et al.* (2022), we implemented a mass univariate approach, which included threshold free cluster enhancement and permutation testing to analyze the spatial (all channels) and temporal (all time points) structure of theta power. A robust increase in theta was observed, peaking at ∼360 ms, which was maximal at frontal midline channels in the 200–500 ms post-response window that corresponds to the N2/P3 ERP complex. We averaged total theta power (4–8 Hz) across the three frontal midline channels (19, 40 and 8 on our montage, approximately corresponding to FCz/Fz on the standard 10–20 montage) with the maximal NOGO-GO effect as our ROI and extracted total peak theta power in the 200–500 ms window to allow for individual variation in the timing of the NOGO theta effect (see van Noordt *et al.*, 2022 for full descriptions of theta classification). A difference score (NOGO-GO) was created to investigate whether youth had greater MF theta power to NOGO compared to GO trials. For clarity, we use the term MF differentiation to mean greater theta NOGO compared to GO. Higher (positive) scores reflect having greater MF theta differentiation (i.e. to NOGO than to GO trials).

Of note, there has also been research suggesting that *right* frontal (RF) activation is linked to threat sensitivity and dimensions of anxiety (Shackman *et al.*, 2009; Neo *et al.*, 2011; Shadli *et al.*, 2021). For example, Neo and McNaughton (2011) found that RF theta was increased during both conflict and loss conditions. In order to test the specificity of MF theta, we also investigated whether RF theta differentiation (NOGO-GO) predicts the intercept and slope of trajectories of threat sensitivity. We extracted theta from a RF channel cluster that approximates locations that correspond to F4/F6 in the standard 10–20 montage.

### Plan of analysis

First, we replicated the latent class growth curve analysis of Heffer and Willoughby (2020b) with a larger sample (432 participants compared to 361 participants). This analysis allows for investigation of whether there are distinct trajectories of self-reported ST across 3 years. Thus, we are able to differentiate youth who have stable and consistently high ST compared to those who report lower or less stable ST. The analysis was conducted using Mplus 7 (Muthén and Muthén, 2012). We used MplusAutomation (Hallquist and Wiley, 2018), a package in R (R Core Team, 2019), to automate the latent class growth curve analysis and extract the model parameters. ST was measured at all three time points and used as latent class indicators. In order to determine the number of trajectories that were best represented by the data, four criteria were considered: (i) interpretability of the classes, (ii) Bayesian information criterion (BIC), such that smaller values of BIC indicate a better fit model, (iii) significance of the Lo-Mendell-Rubin likelihood ratio test (LMR-LRT) significance value—once non-significance is reached, the number of classes prior to non-significance is defined as the appropriate number and (iv) average latent class conditional probabilities are ∼1.00 (Nylund *et al.*, 2007). Entropy (an index of confidence that individuals belong to the correct class and that adequate separation between latent classes exists) was also examined; although there is no set cut-off criterion, entropy scores >0.80 are considered good (Jung and Wickrama, 2008).

**Fig. 1.**
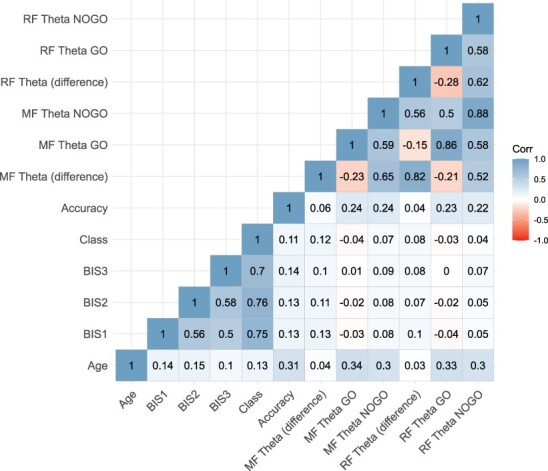
Correlation plot between study variables. Numbers 1, 2 and 3 represent the year.

After establishing the latent trajectories, an ANCOVA was run to investigate class differences on MF theta differentiation (difference score of NOGO-GO as the dependent variable) during response inhibition. NOGO accuracy and age were included as covariates. Given that latent class analyses assume that every participant in a given trajectory class has the same degree of threat sensitivity, we also examined threat sensitivity trajectories continuously to ensure that the pattern of results found using distinct classes replicates. Thus, we ran a growth curve analysis with ST across 3 years. The MF theta difference score, age and accuracy were included as predictors of both the slope and intercept. Finally, to test the specificity of trajectories of threat sensitivity and MF theta, we replicated our above-mentioned analysis using RF theta differentiation. Figure 1 shows correlation table of all study variables.

## Results

### Latent class growth curve analysis

**Fig. 2.**
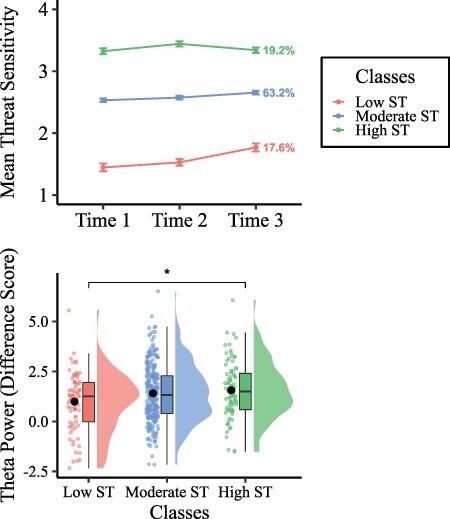
*The top panel* shows the results of the latent class growth curve analysis. Prevalence rates are presented for each class. *The bottom panel* is a raincloud plot showing the distribution of MF theta power for each of the latent classes. Dots represent the raw scores for each class (with jitter added so that the scores are not directly on top of each other), and the mean is depicted as the black dot. To the right of the raw scores, a box plot is presented, with the middle line reflecting the median score. Each class’s distribution for theta power is presented to the right of the box plots. Of note, the skewness for the theta difference score is 0.23. When split by class, the skewness for the theta difference score ranges from −0.14 to 0.33. *P < 0.05.

The latent class growth curve analysis was conducted for 1–5 classes. Consistent with Heffer and Willoughby (2020b), the three-class solution represented the best classification (Table 1). The three-class solution was chosen because the largest decrease in the BIC value was from two to three classes, and the LMR significance value was significant at three classes but not at four classes, suggesting that three classes were a better fit for the data. This solution also was interpretable, replicated previous results and had conditional probabilities ∼1.00. The three trajectories were labeled as low ST (17.6% of the sample), moderate ST (63.2% of the sample) and high ST (19.2% of the sample). Note that these classes appear to differ primarily in degree (e.g. low, moderate and high) rather than kind. Figure 2 shows illustration of the trajectories. The means for threat sensitivity across all three time points for each trajectory class, and the slopes, are presented in Table 2.

**Table 1.** Latent class fit indices

Classes	BIC	Entropy	Conditional probabilities	LMR significance	BLRT significance
2 Classes	2625.330	0.792	0.848–0.975	0.000	<0.001
**3 Classes**	**2558.040**	**0.725**	**0.728–0.915**	**0.030**	**<0.001**
4 Classes	2556.714	0.786	0.684–0.920	0.080	<0.001
5 Classes	2551.801	0.776	0.711–0.940	0.060	<0.001

Note. BLRT = Bootstrapped LRT. The three-class solution (bolded) represented the best classification.

**Table 2.** Class means on sensitivity to threat and their slopes

Variable	Low ST	Moderate ST	High ST	Overall ST
Mean 1 (s.d.)	1.446 (0.544)	2.531 (0.491)	3.323 (0.449)	2.492 (0.754)
Mean 2 (s.d.)	1.528 (0.473)	2.574 (0.518)	3.443 (0.398)	2.557 (0.759)
Mean 3 (s.d)	1.769 (0.564)	2.655 (0.487)	3.340 (0.403)	2.630 (0.681)
Slope (SE)	0.144 (0.096)	0.058 (0.029)*	0.036 (0.055)	

Note. Means 1, 2, and 3 represent the means at Years 1, 2 and 3 of the study, respectively. *P < 0.05.

### Differences among classes on theta power

**Fig. 3.**
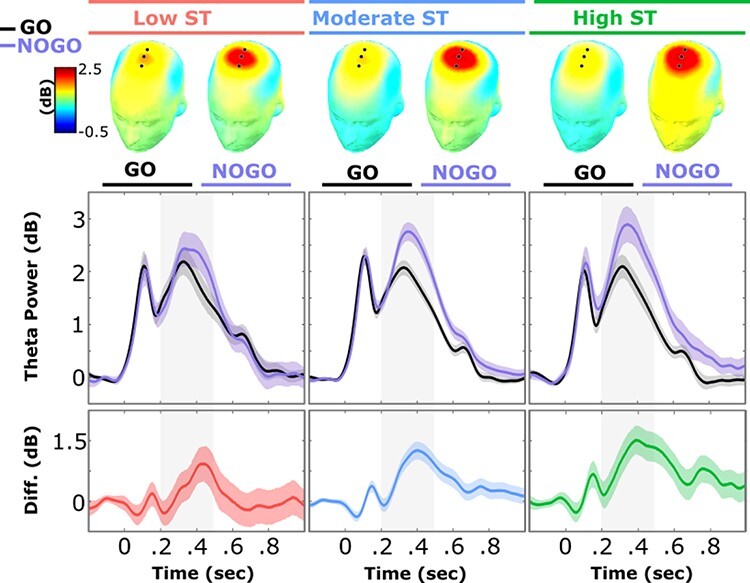
The top of the figure shows topographical maps for theta power for GO and NOGO trials, separated by the identified latent classes. Fronto-central midline channels with maximum theta difference are overlaid on topographical maps. The theta power panel shows theta condition difference for each latent class. Shaded bands around waveforms represent 95% bootstrapped confidence intervals of the mean at each time point. Note that the smaller interval for moderate ST is due to their larger *N*. Gray-shaded regions highlight the time window (200–500 ms) used to extract theta and generate topographical maps. Bottom panel shows mean differences between the theta conditions for each latent class.

An ANCOVA was run to investigate class (low, moderate and high ST) differences on MF theta differentiation (difference score of NOGO-GO) during response inhibition. NOGO accuracy and age were included as covariates. There was a significant main effect of class *F*(2,427) = 3.133, *P* = 0.044, η_p_^2^ = 0.01, a small effect. Follow-up analyses revealed that the high ST class (*M* = 1.56, s.d. = 1.43) had higher MF theta differentiation in comparison to the low ST class (*M* = 0.989, s.d. = 1.45; *P* = 0.031, Cohen’s *d* = 0.396, a small effect). A trend was found whereby the moderate ST class (*M* = 1.41, s.d. = 1.41) had higher MF theta differentiation than the low ST class, *P* = 0.059, Cohen’s *d* = 0.297. The moderate ST class did not differ from the high ST class, *P* = 0.677. Figure 3 shows a visual illustration of distributions and mean scores on theta power across class. NOGO accuracy and age were not significantly different in terms of MF theta differentiation.

Of note, although exploring difference scores (i.e. NOGO-GO) can specifically examine whether individuals have higher theta response to NOGO compared to GO, they are not able to differentiate between individuals who have high MF theta in response to both NOGOs and GOs and individuals who have low MF theta to both NOGOs and GOs (i.e. both cases would have similar difference scores). Thus, we also conducted an ANCOVA assessing whether the classes differed on NOGO theta while controlling for GO theta (and vice versa). NOGO theta in this case was the dependent variable, class was the independent variable and GO theta was a covariate (we also reran the analysis with GO as the dependent variable and NOGO theta as the covariate). Our results remained consistent. That is, class was a significant predictor of NOGO theta, *F*(2,428) = 3.282, *P* = 0.038, η_p_^2^ = 0.02, a small effect, but not a significant predictor of GO theta, *F*(2,428) = 2.307, *P* = 0.101.

Given that latent class analyses assume that every participant in a given trajectory class has the same degree of threat sensitivity, we also ran a growth curve analysis with continuous measures of ST across 3 years. The MF theta difference score, age and accuracy were included as predictors of both the slope and intercept of ST. Table 3 presents the results. The overall model fit was excellent, CFI = 1, RMSEA < 0.001. Greater MF theta differentiation (NOGO-GO) and older age predicted a higher intercept of ST (*Ps* < 0.05). Accuracy was not a significant predictor of the intercept of ST (*P* = 0.09). None of the variables significantly predicted the slope of ST.

**Table 3.** Growth curve results

Variable	Intercept M (SE)	Slope M (SE)
Theta difference	0.063 (0.24)**	−0.009 (0.012)
Age	0.049 (0.02)**	−0.012 (0.010)
Accuracy	0.459 (0.27)	0.050 (0.135)

Note. *P < 0.05; **P < 0.01; ***P < 0.001.

### Threat sensitivity and RF theta

An ANCOVA was run to investigate class (low, moderate, and high ST) differences on the RF theta (difference score of NOGO-GO) during response inhibition. NOGO accuracy and age were included as covariates. There were no significant differences, class: *F*(2,427) = 1.289, *P* = 0.277; age: *F*(2,427) = 0.089, *P* = 0.765 and accuracy: *F*(2,427) = 0.238, *P* = 0.626. We also conducted an ANCOVA assessing whether the classes differed on right NOGO theta while controlling for right GO theta (and vice versa). Right NOGO theta in this case was the dependent variable, class was the independent variable and right GO theta was a covariate (we also reran the analysis with GO as the dependent variable and NOGO theta as the covariate). Our results remained consistent. That is, when we reran the model using right theta NOGO (controlling for GO and vice versa), class was again not a significant predictor of RF NOGO theta, *F*(2,428) = 1.304, *P* = 0.272 or GO theta, *F*(2,428) = 1.018, *P* = 0.362.

## Discussion

The goal of the current study was to assess whether a neural indicator of cognitive control, MF theta differentiation, was associated with longitudinal trajectories of sensitivity to threat. MF theta dynamics represent a common neural signature of the instantiation of cognitive control and performance monitoring (Cavanagh and Frank, 2014; Oehrn *et al.*, 2014). Hypervigilant performance monitoring is thought to be an important behavioral characteristic of individuals with heightened sensitivity to threat, and thus, understanding the dynamic interplay between hypervigilant performance monitoring and sensitivity to threat may be important to identify youth who are most at risk for anxiety. Using a large-scale EEG sample of children and adolescents, we demonstrated that youth with consistently high sensitivity to threat over time were characterized by greater differentiation (i.e. more sensitivity to NOGO than GO trials) of MF theta during response inhibition than youth with consistently low sensitivity to threat over time. Our results provide support for the notion that hypervigilant performance monitoring is an important characteristic associated with threat sensitivity.

These findings are in line with past research linking MF theta dynamics to anxiety and threat processing (e.g. Neo and McNaughton, 2011; Cavanagh and Shackman, 2015; van Noordt *et al.*, 2015). In the context of adaptive behavioral control, these frontal midline theta dynamics may reflect an increased sensitivity to monitoring and detecting threats, perhaps to rapidly facilitate escape or avoidance of challenging and potentially aversive outcomes (van Noordt *et al.*, 2018). Indeed, generalized anxiety has been linked to enhanced performance monitoring (i.e. detection of response errors) and conflict detection (i.e. stimulus–response incongruence) as reflected by elevated MF theta-band activity (Cavanagh *et al.*, 2017). We add to this line of research by showing that developmental trajectories of threat sensitivity over time predict MF theta differentiation (NOGO-GO) during response inhibition.

We did not find, however, that greater differentiation (i.e. more sensitivity to NOGO than GO trials) of MF theta during response inhibition was associated with age or accuracy. Although this was surprising, the bivariate correlations between these variables and theta were small but in the expected direction (i.e. higher theta was associated with better accuracy and older age). Of note, our previous work investigating developmental trajectories of threat sensitivity (Heffer and Willoughby, 2020b) found that individuals with more advanced pubertal development had greater odds of being in high sensitivity to threat class compared to other classes. In other words, adolescents are more likely to be in this high sensitivity to threat class, and thus, it may be that the variance that is typically accounted for by accuracy and age is accounted for by class membership.

In an additional analysis, we also wanted to investigate whether our results remained consistent when we investigated sensitivity to threat trajectories continuously, as opposed to separated by classes. We found that MF theta differentiation predicted the intercept of the sensitivity to threat trajectory, but not the slope. Specifically, a more positive MF theta difference score (i.e. greater MF theta to NOGO than GO trials) was associated with a higher initial threat sensitivity score at Year 1. The difference score was not associated with change in sensitivity to threat across years. This result is consistent with the results found from our latent class analysis, given that the classes were mainly differentiated by degree (low, moderate and high), not by their slopes.

In order to test the specificity of our findings, we also assessed whether trajectories of sensitivity to threat were associated with right frontal (RF) theta differentiation. Although there has been work suggesting that anxiety-related problems are related to RF activation (Shackman *et al.*, 2009; Shadli *et al.*, 2021), our results did not support this conclusion. RF theta differentiation was not associated with trajectories of threat sensitivity among our sample of children and adolescents. This difference in findings likely is a result of different methodologies and samples. For example, Shackman *et al.* (2009) investigated RF alpha activation during rest among university students, and Shadli *et al.* (2021) used a clinically anxious sample and used a complex two-factor contrast to capture pure conflict-related theta.

There are several limitations of this study. First, the full BIS was not included. As the data were part of a larger study assessing a wide range of constructs, it was not feasible to include every item from each scale due to time constraints. Nonetheless, the alpha for this scale across 3 years demonstrated good reliability (ranging from 0.77 to 0.80 across the 3 years; Cronbach, 1951). Second, EEG data were not collected at each time point; thus, we were unable to capture trajectories of theta dynamics over time. Future research should investigate the longitudinal trajectories of both threat sensitivity and theta. In our sample, ∼16% of our sample had negative difference score values. This indicates that these participants had larger MF theta to GO than to NOGO trials. Of note, the majority of these participants had scores hovering around zero (∼70% of the individuals with a negative difference score had a value >−1). Thus, the magnitude of this effect was not very large. While we were interested in MF theta dynamics in relation to threat sensitivity, it is important to acknowledge that theta frequencies have been linked to a wide number of processes (e.g. movement) in humans and animals and thus should not be considered solely in the context of threat sensitivity (e.g. Mitchell *et al.*, 2008). Finally, future research should assess whether longitudinal trajectories of threat sensitivity and MF theta predict anxiety.

The current study offers an important contribution to the literature on theta, performance monitoring and anxiety-related processes. We demonstrate that youth with consistently high threat sensitivity across 3 years have greater MF theta differentiation than youth with consistently low threat sensitivity over time. Hypervigilant performance monitoring may be an important factor to consider when identifying groups of youth at risk for anxiety-related problems, and MF theta dynamics may be a useful neural indicator to aid in this process.
